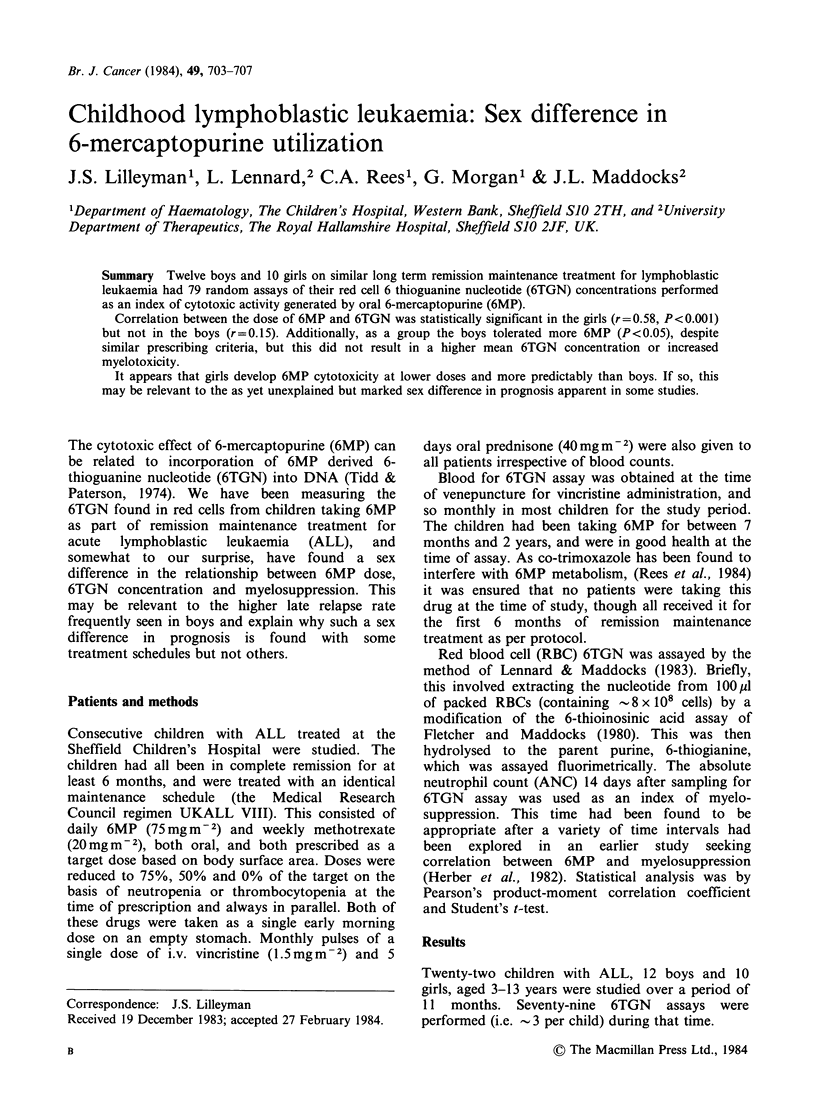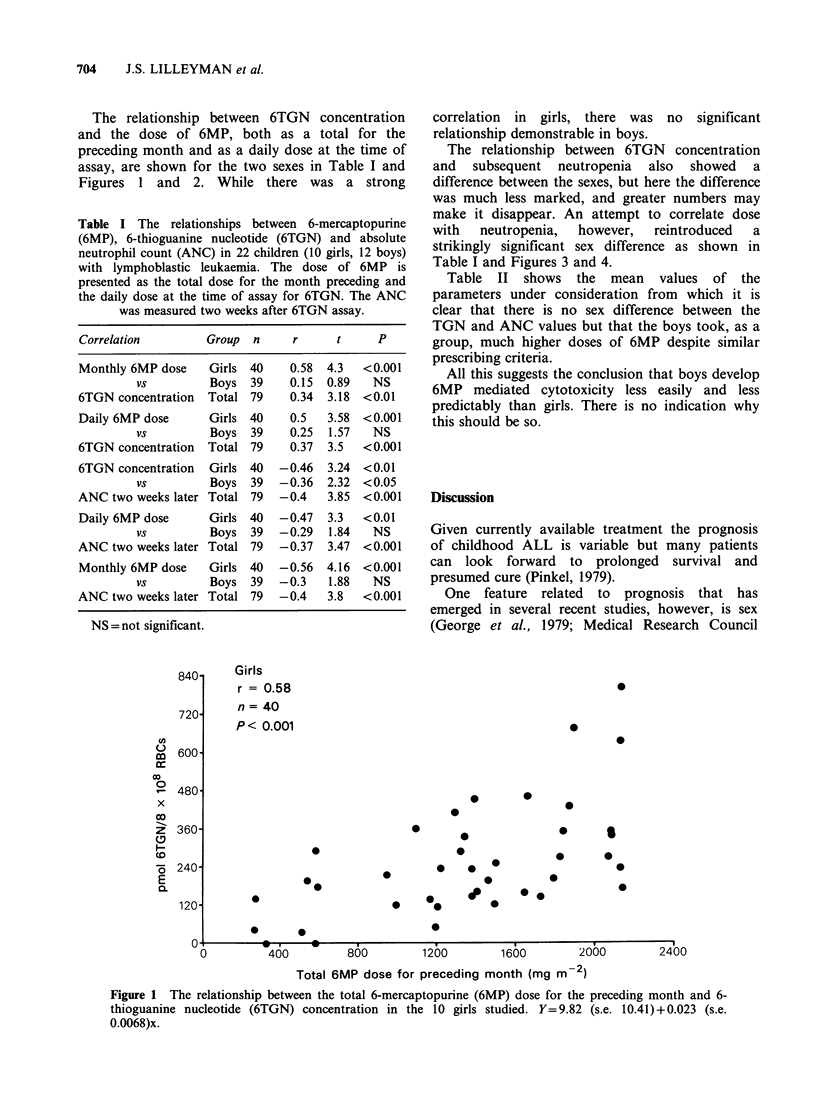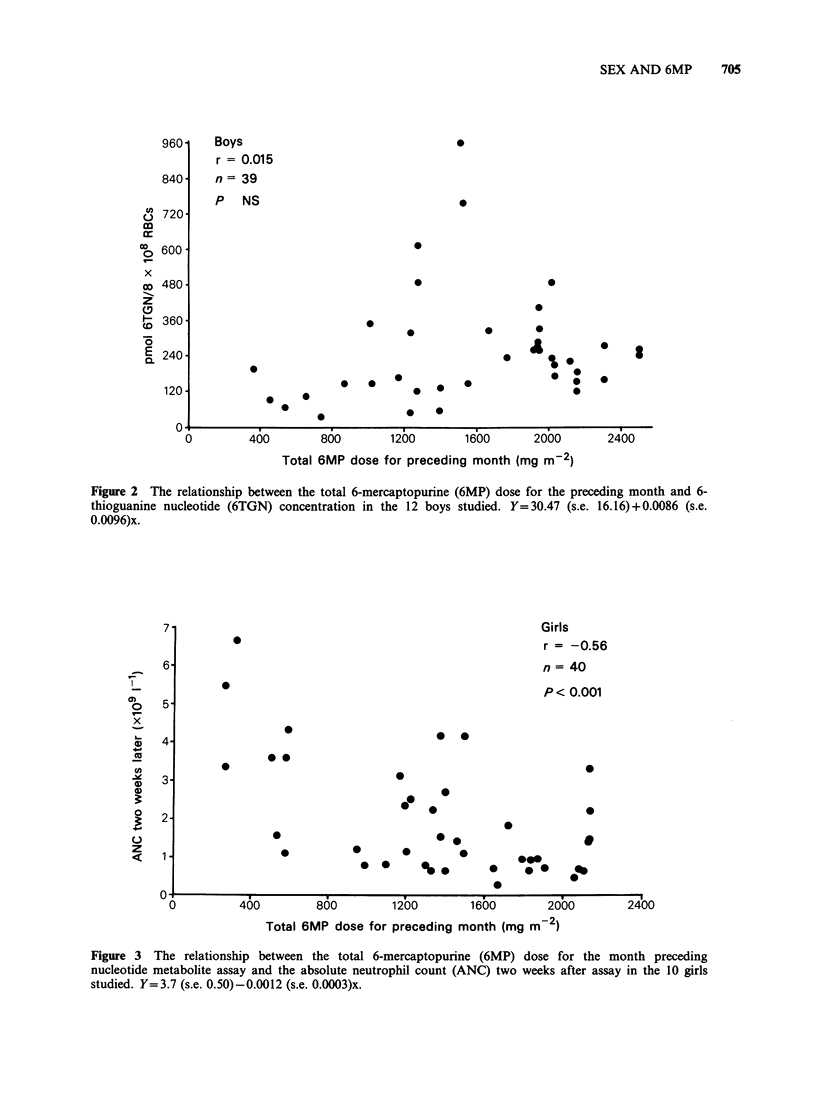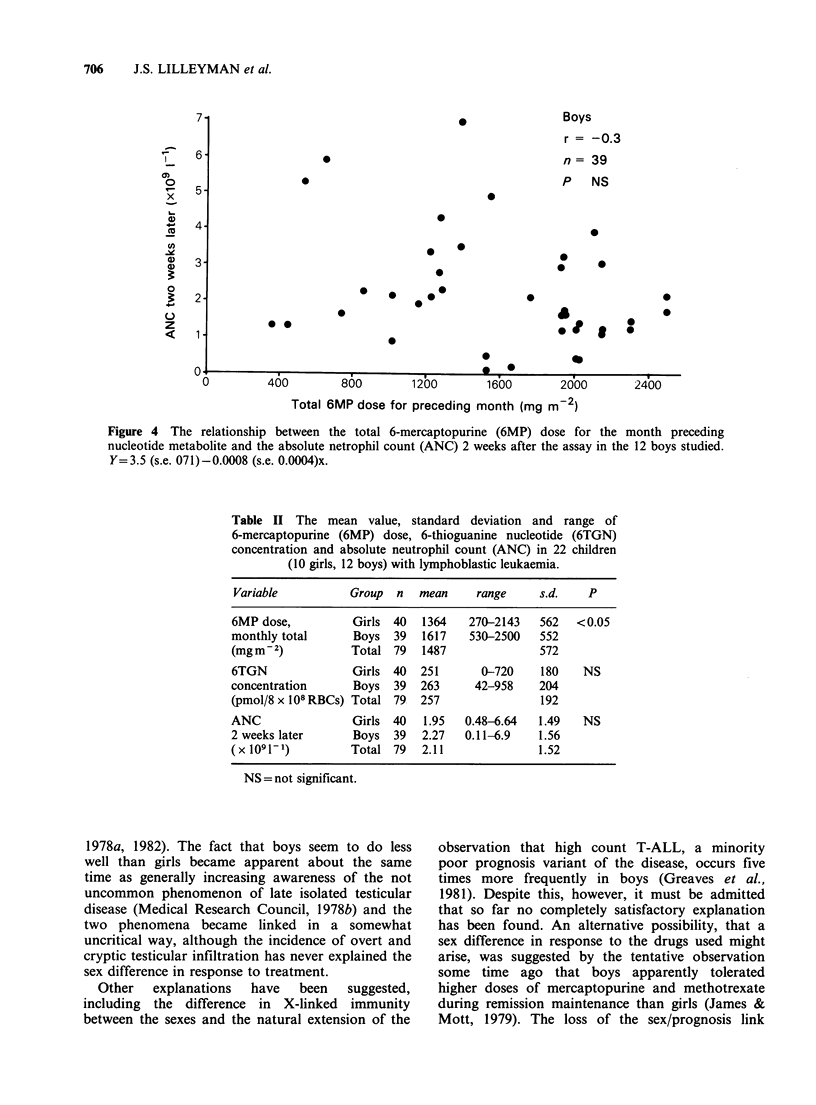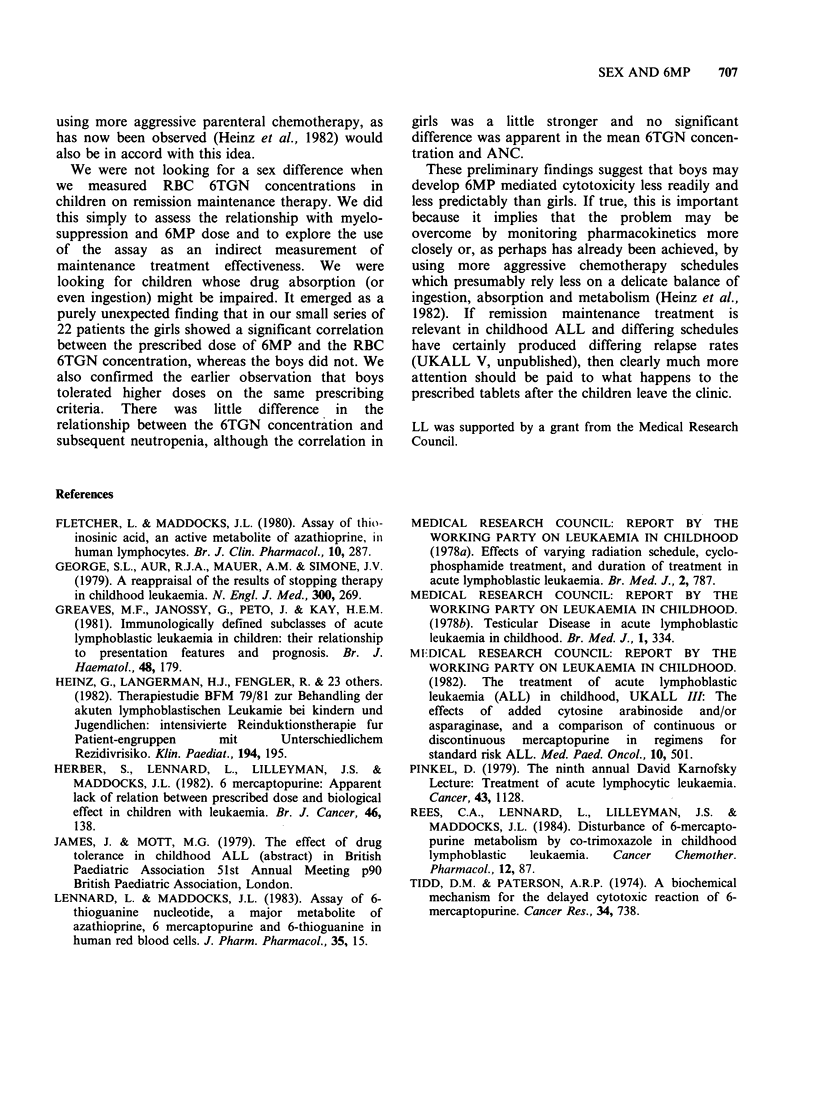# Childhood lymphoblastic leukaemia: sex difference in 6-mercaptopurine utilization.

**DOI:** 10.1038/bjc.1984.111

**Published:** 1984-06

**Authors:** J. S. Lilleyman, L. Lennard, C. A. Rees, G. Morgan, J. L. Maddocks

## Abstract

Twelve boys and 10 girls on similar long term remission maintenance treatment for lymphoblastic leukaemia had 79 random assays of their red cell 6 thioguanine nucleotide ( 6TGN ) concentrations performed as an index of cytotoxic activity generated by oral 6-mercaptopurine ( 6MP ). Correlation between the dose of 6MP and 6TGN was statistically significant in the girls (r = 0.58, P less than 0.001) but not in the boys (r = 0.15). Additionally, as a group the boys tolerated more 6MP (P less than 0.05), despite similar prescribing criteria, but this did not result in a higher mean 6TGN concentration or increased myelotoxicity. It appears that girls develop 6MP cytotoxicity at lower doses and more predictably than boys. If so, this may be relevant to the as yet unexplained but marked sex difference in prognosis apparent in some studies.


*Br. J. Cancer* (1984), **49**, 703–707

### Childhood lymphoblastic leukaemia: Sex difference in 6-mercaptopurine utilization

J.S. Lilleyman[1], L. Lennard,[2] C.A. Rees[1], G. Morgan[1] & J.L. Maddocks[2]

[1]*Department of Haematology, The Children's Hospital, Western Bank, Sheffield S10 2TH, and* [2]*University Department of Therapeutics, The Royal Hallamshire Hospital, Sheffield S10 2JF, UK.*

**Summary** Twelve boys and 10 girls on similar long term remission maintenance treatment for lymphoblastic leukaemia had 79 random assays of their red cell 6 thioguanine nucleotide (6TGN) concentrations performed as an index of cytotoxic activity generated by oral 6-mercaptopurine (6MP).

Correlation between the dose of 6MP and 6TGN was statistically significant in the girls ($r=0.58$, $P<0.001$) but not in the boys ($r=0.15$). Additionally, as a group the boys tolerated more 6MP ($P<0.05$), despite similar prescribing criteria, but this did not result in a higher mean 6TGN concentration or increased myelotoxicity.

It appears that girls develop 6MP cytotoxicity at lower doses and more predictably than boys. If so, this may be relevant to the as yet unexplained but marked sex difference in prognosis apparent in some studies.

The cytotoxic effect of 6-mercaptopurine (6MP) can be related to incorporation of 6MP derived 6-thioguanine nucleotide (6TGN) into DNA (Tidd & Paterson, 1974). We have been measuring the 6TGN found in red cells from children taking 6MP as part of remission maintenance treatment for acute lymphoblastic leukaemia (ALL), and somewhat to our surprise, have found a sex difference in the relationship between 6MP dose, 6TGN concentration and myelosuppression. This may be relevant to the higher late relapse rate frequently seen in boys and explain why such a sex difference in prognosis is found with some treatment schedules but not others.

### Patients and methods

Consecutive children with ALL treated at the Sheffield Children's Hospital were studied. The children had all been in complete remission for at least 6 months, and were treated with an identical maintenance schedule (the Medical Research Council regimen UKALL VIII). This consisted of daily 6MP ($75\,mg\,m^{-2}$) and weekly methotrexate ($20\,mg\,m^{-2}$), both oral, and both prescribed as a target dose based on body surface area. Doses were reduced to 75%, 50% and 0% of the target on the basis of neutropenia or thrombocytopenia at the time of prescription and always in parallel. Both of these drugs were taken as a single early morning dose on an empty stomach. Monthly pulses of a single dose of i.v. vincristine ($1.5\,mg\,m^{-2}$) and 5 days oral prednisone ($40\,mg\,m^{-2}$) were also given to all patients irrespective of blood counts.

Blood for 6TGN assay was obtained at the time of venepuncture for vincristine administration, and so monthly in most children for the study period. The children had been taking 6MP for between 7 months and 2 years, and were in good health at the time of assay. As co-trimoxazole has been found to interfere with 6MP metabolism, (Rees *et al.*, 1984) it was ensured that no patients were taking this drug at the time of study, though all received it for the first 6 months of remission maintenance treatment as per protocol.

Red blood cell (RBC) 6TGN was assayed by the method of Lennard & Maddocks (1983). Briefly, this involved extracting the nucleotide from $100\,\mu l$ of packed RBCs (containing $\sim 8\times10^8$ cells) by a modification of the 6-thioinosinic acid assay of Fletcher and Maddocks (1980). This was then hydrolysed to the parent purine, 6-thiogianine, which was assayed fluorimetrically. The absolute neutrophil count (ANC) 14 days after sampling for 6TGN assay was used as an index of myelosuppression. This time had been found to be appropriate after a variety of time intervals had been explored in an earlier study seeking correlation between 6MP and myelosuppression (Herber *et al.*, 1982). Statistical analysis was by Pearson's product-moment correlation coefficient and Student's *t*-test.

### Results

Twenty-two children with ALL, 12 boys and 10 girls, aged 3–13 years were studied over a period of 11 months. Seventy-nine 6TGN assays were performed (i.e. $\sim 3$ per child) during that time.

Correspondence: J.S. Lilleyman
Received 19 December 1983; accepted 27 February 1984.

© The Macmillan Press Ltd., 1984

The relationship between 6TGN concentration and the dose of 6MP, both as a total for the preceding month and as a daily dose at the time of assay, are shown for the two sexes in Table I and Figures 1 and 2. While there was a strong correlation in girls, there was no significant relationship demonstrable in boys.

**Table I** The relationships between 6-mercaptopurine (6MP), 6-thioguanine nucleotide (6TGN) and absolute neutrophil count (ANC) in 22 children (10 girls, 12 boys) with lymphoblastic leukaemia. The dose of 6MP is presented as the total dose for the month preceding and the daily dose at the time of assay for 6TGN. The ANC was measured two weeks after 6TGN assay.

| *Correlation* | *Group* | *n* | *r* | *t* | *P* |
|---|---|---|---|---|---|
| Monthly 6MP dose | Girls | 40 | 0.58 | 4.3 | <0.001 |
| *vs* | Boys | 39 | 0.15 | 0.89 | NS |
| 6TGN concentration | Total | 79 | 0.34 | 3.18 | <0.01 |
| Daily 6MP dose | Girls | 40 | 0.5 | 3.58 | <0.001 |
| *vs* | Boys | 39 | 0.25 | 1.57 | NS |
| 6TGN concentration | Total | 79 | 0.37 | 3.5 | <0.001 |
| 6TGN concentration | Girls | 40 | −0.46 | 3.24 | <0.01 |
| *vs* | Boys | 39 | −0.36 | 2.32 | <0.05 |
| ANC two weeks later | Total | 79 | −0.4 | 3.85 | <0.001 |
| Daily 6MP dose | Girls | 40 | −0.47 | 3.3 | <0.01 |
| *vs* | Boys | 39 | −0.29 | 1.84 | NS |
| ANC two weeks later | Total | 79 | −0.37 | 3.47 | <0.001 |
| Monthly 6MP dose | Girls | 40 | −0.56 | 4.16 | <0.001 |
| *vs* | Boys | 39 | −0.3 | 1.88 | NS |
| ANC two weeks later | Total | 79 | −0.4 | 3.8 | <0.001 |

NS = not significant.

The relationship between 6TGN concentration and subsequent neutropenia also showed a difference between the sexes, but here the difference was much less marked, and greater numbers may make it disappear. An attempt to correlate dose with neutropenia, however, reintroduced a strikingly significant sex difference as shown in Table I and Figures 3 and 4.

Table II shows the mean values of the parameters under consideration from which it is clear that there is no sex difference between the TGN and ANC values but that the boys took, as a group, much higher doses of 6MP despite similar prescribing criteria.

All this suggests the conclusion that boys develop 6MP mediated cytotoxicity less easily and less predictably than girls. There is no indication why this should be so.

### Discussion

Given currently available treatment the prognosis of childhood ALL is variable but many patients can look forward to prolonged survival and presumed cure (Pinkel, 1979).

One feature related to prognosis that has emerged in several recent studies, however, is sex (George *et al.*, 1979; Medical Research Council

**Figure 1** The relationship between the total 6-mercaptopurine (6MP) dose for the preceding month and 6-thioguanine nucleotide (6TGN) concentration in the 10 girls studied. $Y = 9.82$ (s.e. 10.41) $+ 0.023$ (s.e. 0.0068)x.

**Figure 2** The relationship between the total 6-mercaptopurine (6MP) dose for the preceding month and 6-thioguanine nucleotide (6TGN) concentration in the 12 boys studied. $Y=30.47$ (s.e. 16.16)$+0.0086$ (s.e. 0.0096)x.

**Figure 3** The relationship between the total 6-mercaptopurine (6MP) dose for the month preceding nucleotide metabolite assay and the absolute neutrophil count (ANC) two weeks after assay in the 10 girls studied. $Y=3.7$ (s.e. 0.50)$-0.0012$ (s.e. 0.0003)x.

**Figure 4** The relationship between the total 6-mercaptopurine (6MP) dose for the month preceding nucleotide metabolite and the absolute netrophil count (ANC) 2 weeks after the assay in the 12 boys studied. $Y=3.5$ (s.e. 071) $-0.0008$ (s.e. 0.0004)x.

**Table II** The mean value, standard deviation and range of 6-mercaptopurine (6MP) dose, 6-thioguanine nucleotide (6TGN) concentration and absolute neutrophil count (ANC) in 22 children (10 girls, 12 boys) with lymphoblastic leukaemia.

| *Variable* | *Group* | *n* | *mean* | *range* | *s.d.* | *P* |
|---|---|---|---|---|---|---|
| 6MP dose, | Girls | 40 | 1364 | 270–2143 | 562 | <0.05 |
| monthly total | Boys | 39 | 1617 | 530–2500 | 552 | |
| (mg m$^{-2}$) | Total | 79 | 1487 | | 572 | |
| 6TGN | Girls | 40 | 251 | 0–720 | 180 | NS |
| concentration | Boys | 39 | 263 | 42–958 | 204 | |
| (pmol/8 x $10^8$ RBCs) | Total | 79 | 257 | | 192 | |
| ANC | Girls | 40 | 1.95 | 0.48–6.64 | 1.49 | NS |
| 2 weeks later | Boys | 39 | 2.27 | 0.11–6.9 | 1.56 | |
| (x $10^9$ l$^{-1}$) | Total | 79 | 2.11 | | 1.52 | |

NS = not significant.

1978*a*, 1982). The fact that boys seem to do less well than girls became apparent about the same time as generally increasing awareness of the not uncommon phenomenon of late isolated testicular disease (Medical Research Council, 1978*b*) and the two phenomena became linked in a somewhat uncritical way, although the incidence of overt and cryptic testicular infiltration has never explained the sex difference in response to treatment.

Other explanations have been suggested, including the difference in X-linked immunity between the sexes and the natural extension of the observation that high count T-ALL, a minority poor prognosis variant of the disease, occurs five times more frequently in boys (Greaves *et al.*, 1981). Despite this, however, it must be admitted that so far no completely satisfactory explanation has been found. An alternative possibility, that a sex difference in response to the drugs used might arise, was suggested by the tentative observation some time ago that boys apparently tolerated higher doses of mercaptopurine and methotrexate during remission maintenance than girls (James & Mott, 1979). The loss of the sex/prognosis link using more aggressive parenteral chemotherapy, as has now been observed (Heinz *et al.*, 1982) would also be in accord with this idea.

We were not looking for a sex difference when we measured RBC 6TGN concentrations in children on remission maintenance therapy. We did this simply to assess the relationship with myelosuppression and 6MP dose and to explore the use of the assay as an indirect measurement of maintenance treatment effectiveness. We were looking for children whose drug absorption (or even ingestion) might be impaired. It emerged as a purely unexpected finding that in our small series of 22 patients the girls showed a significant correlation between the prescribed dose of 6MP and the RBC 6TGN concentration, whereas the boys did not. We also confirmed the earlier observation that boys tolerated higher doses on the same prescribing criteria. There was little difference in the relationship between the 6TGN concentration and subsequent neutropenia, although the correlation in girls was a little stronger and no significant difference was apparent in the mean 6TGN concentration and ANC.

These preliminary findings suggest that boys may develop 6MP mediated cytotoxicity less readily and less predictably than girls. If true, this is important because it implies that the problem may be overcome by monitoring pharmacokinetics more closely or, as perhaps has already been achieved, by using more aggressive chemotherapy schedules which presumably rely less on a delicate balance of ingestion, absorption and metabolism (Heinz *et al.*, 1982). If remission maintenance treatment is relevant in childhood ALL and differing schedules have certainly produced differing relapse rates (UKALL V, unpublished), then clearly much more attention should be paid to what happens to the prescribed tablets after the children leave the clinic.

LL was supported by a grant from the Medical Research Council.

### References

FLETCHER, L. & MADDOCKS, J.L. (1980). Assay of thioinosinic acid, an active metabolite of azathioprine, in human lymphocytes. *Br. J. Clin. Pharmacol.*, **10**, 287.

GEORGE, S.L., AUR, R.J.A., MAUER, A.M. & SIMONE, J.V. (1979). A reappraisal of the results of stopping therapy in childhood leukaemia. *N. Engl. J. Med.*, **300**, 269.

GREAVES, M.F., JANOSSY, G., PETO, J. & KAY, H.E.M. (1981). Immunologically defined subclasses of acute lymphoblastic leukaemia in children: their relationship to presentation features and prognosis. *Br. J. Haematol.*, **48**, 179.

HEINZ, G., LANGERMAN, H.J., FENGLER, R. & 23 others. (1982). Therapiestudie BFM 79/81 zur Behandling der akuten lymphoblastischen Leukamie bei kindern und Jugendlichen: intensivierte Reinduktionstherapie fur Patient-engruppen mit Unterschiedlichem Rezidivrisiko. *Klin. Paediat.*, **194**, 195.

HERBER, S., LENNARD, L., LILLEYMAN, J.S. & MADDOCKS, J.L. (1982). 6 mercaptopurine: Apparent lack of relation between prescribed dose and biological effect in children with leukaemia. *Br. J. Cancer*, **46**, 138.

JAMES, J. & MOTT, M.G. (1979). The effect of drug tolerance in childhood ALL (abstract) in British Paediatric Association 51st Annual Meeting p90 British Paediatric Association, London.

LENNARD, L. & MADDOCKS, J.L. (1983). Assay of 6-thioguanine nucleotide, a major metabolite of azathioprine, 6 mercaptopurine and 6-thioguanine in human red blood cells. *J. Pharm. Pharmacol.*, **35**, 15.

MEDICAL RESEARCH COUNCIL: REPORT BY THE WORKING PARTY ON LEUKAEMIA IN CHILDHOOD (1978*a*). Effects of varying radiation schedule, cyclophosphamide treatment, and duration of treatment in acute lymphoblastic leukaemia. *Br. Med. J.*, **2**, 787.

MEDICAL RESEARCH COUNCIL: REPORT BY THE WORKING PARTY ON LEUKAEMIA IN CHILDHOOD. (1978*b*). Testicular Disease in acute lymphoblastic leukaemia in childhood. *Br. Med. J.*, **1**, 334.

MEDICAL RESEARCH COUNCIL: REPORT BY THE WORKING PARTY ON LEUKAEMIA IN CHILDHOOD. (1982). The treatment of acute lymphoblastic leukaemia (ALL) in childhood, UKALL *III*: The effects of added cytosine arabinoside and/or asparaginase, and a comparison of continuous or discontinuous mercaptopurine in regimens for standard risk ALL. *Med. Paed. Oncol.*, **10**, 501.

PINKEL, D. (1979). The ninth annual David Karnofsky Lecture: Treatment of acute lymphocytic leukaemia. *Cancer*, **43**, 1128.

REES, C.A., LENNARD, L., LILLEYMAN, J.S. & MADDOCKS, J.L. (1984). Disturbance of 6-mercaptopurine metabolism by co-trimoxazole in childhood lymphoblastic leukaemia. *Cancer Chemother. Pharmacol.*, **12**, 87.

TIDD, D.M. & PATERSON, A.R.P. (1974). A biochemical mechanism for the delayed cytotoxic reaction of 6-mercaptopurine. *Cancer Res.*, **34**, 738.